# Congenital and neonatal malaria in a rural Kenyan district hospital: An eight-year analysis

**DOI:** 10.1186/1475-2875-9-313

**Published:** 2010-11-06

**Authors:** Michael K Mwaniki, Alison W Talbert, Florence N Mturi, James A Berkley, Piet Kager, Kevin Marsh, Charles R Newton

**Affiliations:** 1 Centre for Geographic Medicine Research (Coast), Kenya Medical Research Institute, Box 230, Kilifi, Kenya; 2 Centre for Clinical Vaccinology and Tropical Medicine, University of Oxford, Churchill Hospital, Oxford, OX3 7LJ, UK; 3 Department of Infectious Diseases, Tropical Medicine and AIDS, Academic Medical Centre, University of Amsterdam, Amsterdam, the Netherlands; 4 Nuffield Department of Clinical Medicine, University of Oxford, John Radcliffe Hospital, Oxford, UK; 5 Clinical Research Unit, London School of Hygiene and Tropical Medicine, London, UK; 6 Neurosciences Unit, Institute of Child Health, London, WC1N 2AP, UK

## Abstract

**Background:**

Malaria remains a significant burden in sub-Saharan Africa. However, data on burden of congenital and neonatal malaria is scarce and contradictory, with some recent studies reporting a high burden. Using prospectively collected data on neonatal admissions to a rural district hospital in a region of stable malaria endemicity in Kenya, the prevalence of congenital and neonatal malaria was described.

**Methods:**

From 1^st ^January 2002 to 31^st ^December 2009, admission and discharge information on all neonates admitted to Kilifi District Hospital was collected. At admission, blood was also drawn for routine investigations, which included a full blood count, blood culture and blood slide for malaria parasites.

**Results:**

Of the 5,114 neonates admitted during the eight-year surveillance period, blood slide for malaria parasites was performed in 4,790 (93.7%). 18 (0.35%) neonates with *Plasmodium falciparum *malaria parasitaemia, of whom 11 were admitted within the first week of life and thus classified as congenital parasitaemia, were identified. 7/18 (39%) had fever. Parasite densities were low, ≤50 per μl in 14 cases. The presence of parasitaemia was associated with low haemoglobin (Hb) of <10 g/dl (χ^2 ^10.9 P = 0.001). The case fatality rate of those with and without parasitaemia was similar. *Plasmodium falciparum *parasitaemia was identified as the cause of symptoms in four neonates.

**Conclusion:**

Congenital and neonatal malaria are rare in this malaria endemic region. Performing a blood slide for malaria parasites among sick neonates in malaria endemic regions is advisable. This study does not support routine treatment with anti-malarial drugs among admitted neonates with or without fever even in a malaria endemic region.

## Background

Malaria remains one of the most significant disease burdens in resource poor countries of sub-Saharan Africa especially among children under the age of five years and pregnant women [1-4]. However, there is some evidence that over the last decade the epidemiology of this disease has changed [5,6]. Importantly, reduction in infection and clinical disease has been reported in several areas [3,6,7]. This could be due to a combination of factors such as increased coverage with insecticide treated mosquito nets and change to a more effective anti-malarial drug [5,8]. In addition, changes in mean age of presentation have been documented [9]. The impact of climate on transmission dynamics and distribution of the vector is increasingly recognized [7].

Malaria contributes significantly to perinatal disease burden in terms of pregnancy losses, prematurity due to preterm labor and intra-uterine growth retardation [10-12]. Despite these well-documented indirect effects of malaria to the fetus and newborn, the direct burden of neonatal malaria infection in terms of prevalence and outcome is not well described in malaria endemic areas. Studies published so far have documented contradictory levels of this burden (See Additional file 1: a summary of key studies describing malaria in the neonatal period) [13-16]. Most of these studies were conducted in large referral or teaching hospitals, and the results are difficult to generalize [13-15,17,18]. Furthermore, many studies lack details of internal and external laboratory quality control procedures, parasite densities, and only examine a small sample of neonates [15,17,19,20]. This limitation further complicates the interpretation and application of the findings.

Given the limitations of the published data, this study describes the prevalence and outcome of congenital and neonatal malaria in all neonates admitted to a rural district hospital in a malaria endemic region in Kenya over an eight-year period from 1^st ^January 2002 to 31^st ^December 2009.

## Methods

### Location

The study was conducted at the Kenya Medical Research Institute (KEMRI) centre located at Kilifi District Hospital on the coast of Kenya, 60 kilometers north of Mombasa. More than seventy percent of neonates admitted to the hospital are born at home in a rural community where the entomological inoculation rate is between 0 and 120 infected bites per year, and the *Plasmodium falciparum *parasite prevalence in children aged 2-10 years ranges from 1.3-25.9 [21,22]. This centre has developed a prospective and continuous inpatient surveillance system aimed at describing the causes and features of common illnesses in a rural resource poor district hospital setting since the 1990s [23,24]. In addition, the hospital is located within the Kilifi epidemiology and demographic surveillance system (EPI-DSS). This is a system whereby a resident population of over 240,000 living within a catchment area of 891 km^2 ^is continuously monitored and vital events updated every four to six months. The entire area was mapped using global positioning system (GPS) and both mapping and population data are linked on-line to the hospital admission data.

Government-employed clinicians in the outpatient department, working independently of the inpatient research team, refer children for admission. In addition, sick neonates are admitted directly from the maternity department. At admission and discharge/death, standardized clinical and laboratory data are prospectively collected by research clinicians and directly entered into a computer database. Consent for use of the data is obtained from the guardian of every child at the point of admission. The Kenya National Scientific and Ethical Review Board approved this study (SCC protocol 1592).

### Study participants and definitions

A neonate was defined as any child admitted aged 28 days or younger [25]. The discharging clinician made the final diagnosis (e.g. neonatal sepsis, neonatal encephalopathy, or prematurity) after review of the admission history, inpatient management notes and laboratory investigations. A supervising clinician counterchecked these diagnoses. Runsewe-Abiodun and colleagues' definition of congenital malaria as symptoms attributable to only malaria with evidence of intra-erythrocytic asexual forms of *Plasmodium *species in the first seven days of life, and neonatal malaria as intra-erythrocytic asexual forms of *Plasmodium *species in the first 28 days of life in a sick neonate was used [15]. Any neonate requiring hospitalization was considered "sick". For other diseases, a definite diagnosis of invasive bacterial disease (IBD) was made upon isolation of pathogenic organisms from blood culture or cerebrospinal fluid (CSF). Otherwise, sepsis was considered as the possible diagnosis in any newborn presenting with any one of the following signs: abnormal temperature (>37.5°C or <35.5°C) and multiple skin pustules, umbilical redness or pus, respiratory distress, lethargy, seizures or feeding problems [26]. Meningitis was defined as a positive CSF culture, or a white cell count of >50/μl in CSF or a positive bacterial antigen test or gram stain [27]. This approach to formulation and definition of the final diagnoses has been previously described in detail [28].

### Admission procedures

At admission, the research clinician performed emergency procedures such as correction of airway, breathing, circulation, hypothermia or hypoglycaemia before taking a formal admission history and physical examination. A venous blood sample was routinely drawn (according to weight but not exceeding 3 mls) for a full blood count, blood slide for malaria parasite examination, blood glucose, electrolytes and microbiological culture. As per World Health Organization (WHO) guidelines, meningitis was considered in all neonates presenting with any one of: drowsiness, lethargy, reduced feeding, high-pitched cry, irritability, apnoeic episodes, a bulging fontanel, unconsciousness or seizures and where not contraindicated, a lumbar puncture was performed [26]. Research microscopists examined malaria slides, and their work was subject to strict quality control procedures both internally with quarterly proficiency tests, and externally as part of a quality control scheme run by the National Institute of Communicable Diseases, South Africa [29]. All neonates admitted received empirical antibiotic treatment (ampicillin and gentamicin). A change of antibiotics and the duration of treatment were guided by the results of culture and the clinical response. Treatment of malaria followed the prevailing national guidelines [30].

### Analysis

Admitting clinicians entered individual patient clinical data at point of contact using a FileMaker Pro database (10.0 v1 Developer, FileMaker Inc, USA). Likewise, laboratory data was double entered and verified using FoxPro for windows (FoxPro 2.5b for Windows, Microsoft Corporation, USA). Pearson's chi square test (or Fisher's exact test as appropriate) was used to compare proportions. Analysis was performed using STATA^® ^11 (Stata Corp, College Station, TX).

## Results

During the study period, a total of 5,114 (59% males) neonates were admitted to Kilifi District Hospital. Most (67%) of the neonates admitted were less than one week old. Blood film results for malaria parasites were available for 4,790 (93.7%) of all neonates: 3,187(92.6%) in the first week of life and 1,606 (96%) thereafter. 18 (0.35%) neonates with a positive blood slide for *P. falciparum *malaria and no neonates with parasitaemia due to other plasmodial species were identified. Eleven of the eighteen were admitted within the first week of life and were thus classified as congenital parasitaemia. Parasite densities were low and in 14 of the cases were fewer than 50 per μl. Three neonates had parasite densities of 2,480, 11,600 and 22,560 per μl respectively and one neonate had 38/500(7.6%) red blood cells parasitized (304,000 parasite per μl). All the cases were admitted between the years 2002 and 2005.

### Clinical presentation, laboratory findings, and final diagnoses of the neonates with parasitaemia

The summary of the clinical and laboratory characteristics for the 18 neonates with parasitaemia is as shown (see additional file 2: Summary of the cases with neonatal parasitaemia admitted to Kilifi District Hospital). Seven (39%) of the neonates had fever (axillary temperature >37.2°C) at admission. The proportions of fever at admission did not differ from those without parasitaemia (χ^2 ^0.049 P = 0.824). Overall the presence of parasitaemia was associated with an Hb of <10 g/dl (χ^2 ^10.9 P = 0.001). Only one case presented with convulsions at admission. The prevalence of hypoglycaemia (blood glucose <2.6 mmols/l) at admission did not differ between the cases with parasitaemia and those without (χ^2 ^0.26 p = 0.61)

Concomitant bacteraemia was found in two of the neonates with malaria parasitemia; one with *Enterobacter *and the other *Acinetobacter*. One neonate had CSF features of meningitis: a white blood cell count of 208 per μl and gram-negative rods on gram stain. The CSF and blood cultures were however negative (case1 additional file 2).

In total, only four of the 18 neonates with parasitaemia were diagnosed to have malaria. These four were immediately treated with anti-malarial (intravenous quinine) drugs in addition to antimicrobial drugs for suspected sepsis. Of the remaining, there were seven diagnosed as neonatal sepsis, four cases of prematurity with severe respiratory distress, two cases of neonatal jaundice and possibly sepsis too, and one case of meningitis (additional file 2). One other neonate with a diagnosis of prematurity with respiratory distress and parasitaemia of 16 per μl was also started on quinine on the 3^rd ^inpatient day when judged not to be improving. This newborn died on the 10^th ^inpatient day (case 7 additional file 2).

### Outcome

Five (27%) of the neonates with parasitaemia died compared to 1,006 (19.7%) of those without parasitaemia (Fisher's exact P = 0.38). Four of the neonates with parasitaemia died within 24 hours of admissions, two possibly from overwhelming sepsis, one from a combination of severe jaundice and possible sepsis and the last from complications of prematurity with respiratory distress. The fifth died on the 10^th ^inpatient day, the likely cause of death been complications of gross prematurity. All the four neonates with high parasitaemia were discharged alive. Follow-up through the demographic surveillance system revealed that all the 13 newborns with parasitaemia at admission, discharged into the community were still alive as of date.

## Discussion

Information on the burden of malaria in early infancy, especially the neonatal period is scarce, inconclusive and contradictory, with a wide range of results reported [13,15,16]. In this study, one of the largest surveillance of neonates in a malaria endemic zone, blood slides for malaria parasites examination were systematically performed for all neonatal admissions. The prevalence of parasitaemia was found to be <0.5% of the neonatal admissions. Overall, a diagnosis of malaria was made in only four of the neonates with parasitaemia.

The result of this study therefore suggests that the burden of neonatal malaria is much less than previously reported. Several single centre studies in Nigeria have reported a parasitaemia prevalence in excess of 20% among sick neonates admitted to different hospitals in the country, and in a sizeable proportion of them, symptomatology attributed to the parasitaemia [15,17,18,20,31]. However, a much larger multicentre study in the same country documented parasitaemia in just 5.1% of neonatal admissions [14]. This finding is similar to a study from Ivory Coast (Cote D'Ivoire), which is almost identical in malaria endemicity to Nigeria, where a prevalence of <1% was reported [32]. Likewise in Malawi, although the burden of malaria in infants under the age of six months was found to range from 2.6-6.7%, the prevalence of parasitaemia was 0.5% among those aged <2 months [16]. High prevalence of congenital and neonatal parasitaemia (>20%) have also been previously reported in Uganda and Zambia [19,33].

The marked differences in prevalence of congenital and neonatal parasitaemia are difficult to explain. Plausible explanations include real epidemiological differences and operational factors. In several recent studies, malaria slides read in routine service have been shown to have very poor positive predictive value when compared to expert microscopy [34,35]. In one centre, only 13% of adults with a diagnosis of cerebral malaria actually had parasitaemia on their blood film [36]. These data suggests that over-diagnosis of malaria may be widespread in sub-Saharan Africa and may lead to under-treatment for other life-threatening conditions such as septicemia. Emphasis should be placed on developing the capacity for rapid and reliable laboratory services with adequate quality control. However, it is also possible that the variations in prevalence of parasitaemia and neonatal malaria between malaria endemic zones may be real, and subtle differences in transmission dynamics and maternal immunity may in part account for these differences [20,31]. Importantly it is worth noting that even among sick neonates with parasitaemia (especially very low levels), it may still prove difficult to assign parasitaemia as the cause of ill health, because these neonates have the same clinical features as those with other causes of neonatal admissions, particularly sepsis. In addition, one study suggests that septic neonates and those with suspected sepsis may be more likely to have parasitaemia [13].

It is worth noting that all the cases of neonatal and congenital malaria were identified in the earlier years (2002-2005). It is plausible that this could partly be explained by the increased drive to combat malaria in the region through a combination of strategies such as increased coverage of insecticide treated mosquito nets, improving antenatal care and uptake of Intermittent preventive Treatment in pregnancy (IPTP), and finally change to a more effective anti-malarial drug that occurred during the decade [5,8]. This finding mirrors the general reduction in the burden of hospital admissions due to malaria in the later part of this decade that has been reported in the region [3,5,8].

A major limitation of this study is that not all neonates with low parasitaemia were treated with antimalaria drugs, although all survived except the ones who died from complications of prematurity or sepsis. It is however worth noting that studies have documented that most cases of such low parasitaemia clear spontaneously without treatment [14,18,31]. It is also worth considering that many cases of low parasitaemia may be false positives. Although microscopic identification of Plasmodium species remains the gold standard for diagnosis of malaria, PCR may offer an attractive addition for confirmatory identification and diagnosis and should be explored in future studies. This may give a more accurate description of the burden of congenital and neonatal malaria [37]. Importantly too, even in facilities located in rural settings in resource poor regions, a sizable proportions of 'sick' neonates may die before any contact with the formal health care system [38]. The study results therefore likely represent the minimum prevalence.

## Conclusions

The results of this study suggest that it is worthwhile to perform blood films on hospitalized neonates with fever in malaria endemic regions since though it is rare, congenital and neonatal malaria does occur. Even where resources are extremely limited, investigation for malaria especially among febrile neonates not responding to standard antibiotics treatment should be undertaken. However, in many instances a positive slide may not explain the cause of illness. Neonates with a positive slide should therefore still receive antimicrobial drugs for sepsis as well as antimalarial drugs. The results however do not support routine treatment with anti-malarial drugs for admitted neonates with or without fever, in this malaria endemic region.

## Competing interests

The authors declare that they have no competing interests.

## Authors' contributions

MKM participated in study design, analyzed the data and drafted the manuscript. AT helped in data analysis and participated in manuscript writing. NM & JB & KM supervised clinical care and participated in manuscript writing. PK assisted in study design and manuscript preparation. CN conceived the study, helped in study design and manuscript writing. All authors read and approved the final manuscript.

## Supplementary Material

Additional file 1 **Table showing a summary of key studies describing malaria in the neonatal period**. Click here for file

Additional file 2 **Table Summary of the cases with neonatal parasitaemia admitted to Kilifi District Hospital**. Click here for file
