# Supplementary material for: Congenital and neonatal malaria in a rural Kenyan district hospital: An eight-year analysis
Source: Malar J. 2010 Nov 6;9:313. doi: 10.1186/1475-2875-9-313 (PMC2988044; doi:10.1186/1475-2875-9-313)
Supplement: Additional file 1 — Table showing a summary of key studies describing malaria in the neonatal period. [file 1475-2875-9-313-S1.DOC]

Table summary of key studies describing malaria in the neonatal period

| study | Year | Country | Institution type | Neonates seen  N (% slide done) | Parasitaemia  N (%) | No. with illness  due to parasitaemia  N(%) | Clinical & laboratory features | | | |
| --- | --- | --- | --- | --- | --- | --- | --- | --- | --- | --- |
|  |  |  |  |  |  |  | fever | pallor | anaemia | bacteraemia |
| Akindele | 1991 | Nigeria | Teaching hospital | 59 (100) | 14(23.7) | - | - | - | - | - |
| Obiajunwa et al. | 1997 | Nigeria | Teaching hospital | 120 (100) | 56(46.7) | - | 2 | - | - | - |
| Runsewe et al. | 1998-1999 | Nigeria | Teaching hospital | 231 (39) | 57(24.8) | 57(24.8) | 44 | - | 0 | - |
| **Mukhtar et al.** | 2002 | Nigeria | Teaching hospital | 104 (100) | 16(15.3) | - | - | - | - | - |
| Okafor‌ et al. | 2003-2004 | Nigeria | Teaching hospital | 658 (95) | 203(32.5) | 23(3.7) | 17 | - | - | - |
| Falade et al.* | 2007 | Nigeria | Teaching hospitals | 1,875(100) | 95(5.1) | 32(33.7) | 32 | - | - | 0 |
| Ekanem et al. | 2008 | Nigeria | Teaching hospital | 202(100) | 71(35.1) | - | - | - | 30 | 14 |
| Adja | 2005-06 | Ivory  Coast | Teaching hospital | 615 (100) | 6(<1) | - | - | - | - | - |
| Larru et al.** | 1998-2008 | Malawi | Teaching hospital | - | (0.5) | - | - | - | - | - |
| Larkin | 1989 | Zambia | Regional hospital | 65 (100) | 19(29.2) | 7(36.8) | 7 | - | - | - |
| Ndyomugyenyi et al. | 2000 | Uganda | District hospital | 510 (93) | 198(41.8) | - | - | - | - | - |

*Multi-centre: recruited done from four teaching hospitals in different regions within the country

**Study looked at malaria in infants aged <6 months. Data shown here represents the proportions with parasitaemia among those aged <2 months
