# Supplementary material for: Congenital and neonatal malaria in a rural Kenyan district hospital: An eight-year analysis
Source: Malar J. 2010 Nov 6;9:313. doi: 10.1186/1475-2875-9-313 (PMC2988044; doi:10.1186/1475-2875-9-313)
Supplement: Additional file 2 — Table Summary of the cases with neonatal parasitaemia admitted to Kilifi District Hospital. [file 1475-2875-9-313-S2.DOC]

Summary of the cases with neonatal eo00000000000000000000000000000000000000000000000000000000000000000000000000000000000000000000000000000000000000000000000000000parasitaemia admitted to Kilifi District Hospital.

| Cases | Age in days | Inpatient  duration | Weight  (Kg) | Parasite  densities per µl | Clinical & laboratory features | | | | | | | Outcome |
| --- | --- | --- | --- | --- | --- | --- | --- | --- | --- | --- | --- | --- |
|  |  |  |  |  | Fever  history | Temperature on admission  (◦C) | Pallor | Hb  (g/dl) | Convulsion | Bacteraemia | Final  diagnosis |  |
| 1 | 5 | 2 | 2.84 | 16 | y | 39.1 | n | 18.2 | n | n | Meningitis | alive |
| 2 | 2 | 7 | 1.45 | 16 | n | 36.7 | n | 14.9 | n | n | Prematurity* | alive |
| 3 | 13 | 3 | 2.0 | 16 | n | 37.1 | y | 11.8 | n | n | Sepsis | alive |
| 4 | 4 | 5 | 2.10 | 32 | n | 36.8 | n | 18.7 | n | n | Sepsis | alive |
| **5** | 5 | 1 | 1.61 | 16 | y | 39.6 | n | 18.4 | n | y | Sepsis (*Enterobacter*) | died |
| 6 | 0 | 0 | 1.3 | 32 | n | 37.2 | n | 12.3 | n | n | Prematurity | died |
| 7 | 0 | 10 | 1.3 | 16 | n | 36.8 | n | 18.9 | n | y | Prematurity | died |
| 8 | 2 | 4 | 2.95 | 16 | n | 36.8 | n | 16.0 | n | n | Sepsis | alive |
| 9 | 5 | 1 | 2.51 | 48 | y | 37.7 | n | 17.0 | n | n | Sepsis (*Acinetobacter*) | died |
| 10 | 4 | 1 | 2.26 | 32 | n | 36.2 | n | 15.6 | n | n | Jaundice & Sepsis | died |
| 11 | 16 | 2 | 4.8 | 11,600 | y | 38.8 | y | 8.2 | n | n | Malaria | alive |
| 12 | 4 | 4 | 3.14 | 16 | y | 37.2 | n | 14.9 | n | n | Jaundice & Sepsis | alive |
| 13 | 1 | 2 | 3.2 | 304,000 | y | 37.5 | y | 5.9 | y | n | Malaria | alive |
| 14 | 12 | 2 | 1.5 | 16 | n | 36.7 | n | 18.2 | n | n | Prematurity | alive |
| 15 | 14 | 1 | 5.26 | 2,480 | y | 37.5 | y | 8.6 | n | n | Malaria | alive |
| 16 | 23 | 4 | 4.60 | 16 | n | 36.9 | y | 12.2 | n | n | Sepsis | alive |
| 17 | 17 | 5 | 3.42 | 32 | n | 36.6 | n | 14.2 | n | n | Sepsis | alive |
| 18 | 16 | 4 | 4.66 | 22,560 | n | 38.1 | y | 5.7 | n | n | Malaria | alive |

*all cases of prematurity had other complications notably respiratory distress.
